# Supplementary material for: Nanog maintains stemness of Lkb1‐deficient lung adenocarcinoma and prevents gastric differentiation
Source: EMBO Mol Med. 2021 Jan 13;13(3):e12627. doi: 10.15252/emmm.202012627 (PMC7933951; doi:10.15252/emmm.202012627)
Supplement: Supplementary file 1 — Expanded View Figures PDF [file EMMM-13-e12627-s001.pdf]

Expanded View Figures

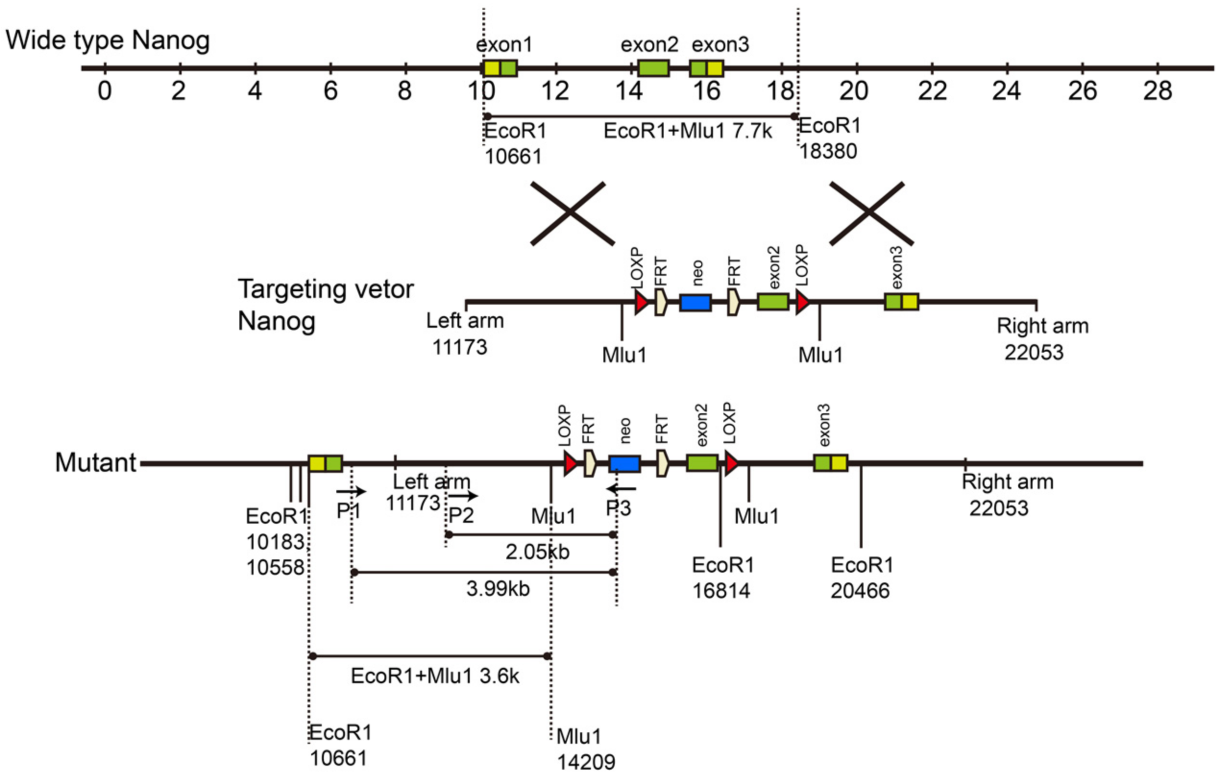

Figure EV1. Schematic illustration of *Nanog*$^{flox/flox}$ mouse model.

To insert LOXP sites in *Nanog*, a homologous recombinant vector was constructed. Targeting vector contained two arms homologous to the genome sequence of the *Nanog* gene, exon2 with LOXP sites on both sides and a drug-screening marker PGK NEO. Mutant allele showing the insertion of LOXP sites on both sides of exon2.

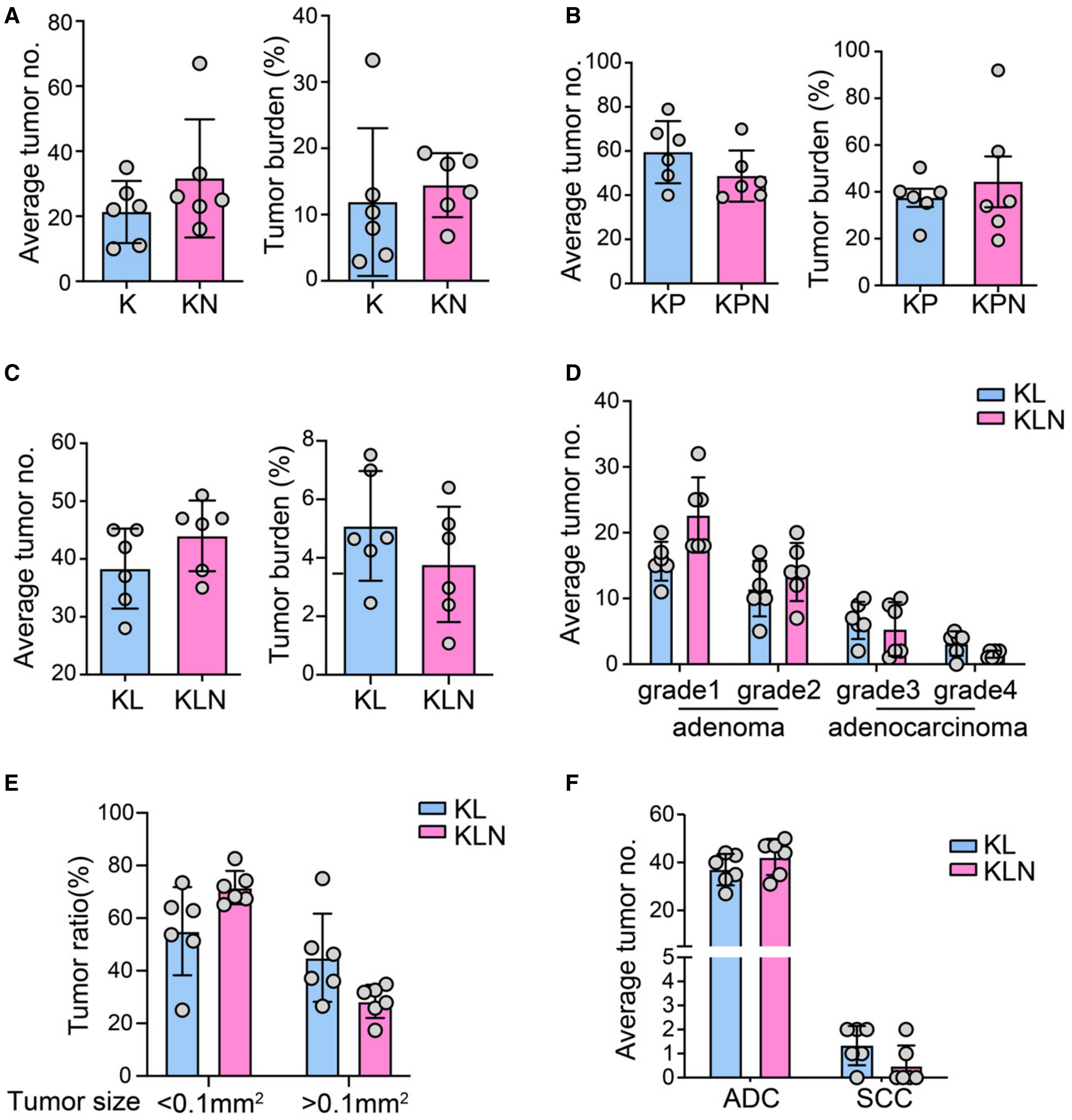

Figure EV2. ***Nanog*** **deletion has little effects upon lung tumorigenesis in** ***Kras***^{G12D}**-driven models.**

A–C Quantification of average tumor number (no.) and tumor burden from indicated mice. Mice were sacrificed after 16 weeks (A, n = 6 per group), 12 weeks (B, n = 6 per group), or 8 weeks (C, n = 6 per group) for pathological analysis.

D Statistical analysis of numbers of tumors with different tumor grade in KL (n = 6) and KLN (n = 6) mice.

E Statistical analysis of ratio of small tumor (<0.1mm^2) and large tumor (>0.1mm^2) in KL (n = 6) and KLN (n = 6) mice.

F Statistical analysis of numbers of ADC and SCC in KL (n = 6) and KLN (n = 6) mice.

© 2021 The Authors

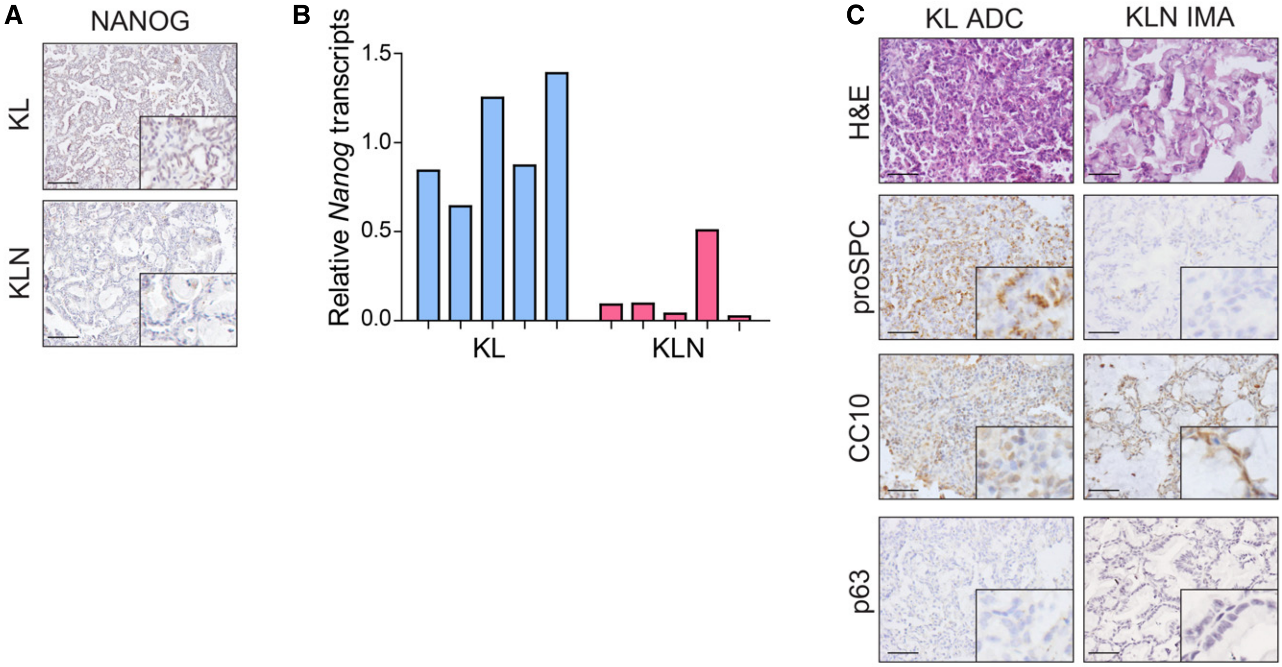

Figure EV3. Simultaneous deletion of *Nanog* and *Lkb1* is indispensable for mucinous tumor emergence in *Kras*^{G12D} mice.

A, B Verification of *Nanog* knockout in mice using IHC staining (A) and qRT–PCR (B) in KL, KLN mice.

C HE and IHC staining of proSPC, p63, and CC10 in mucinous tumors and non-mucinous tumors of KLN mice. Representative images were shown. Scale bar, 50 μm.

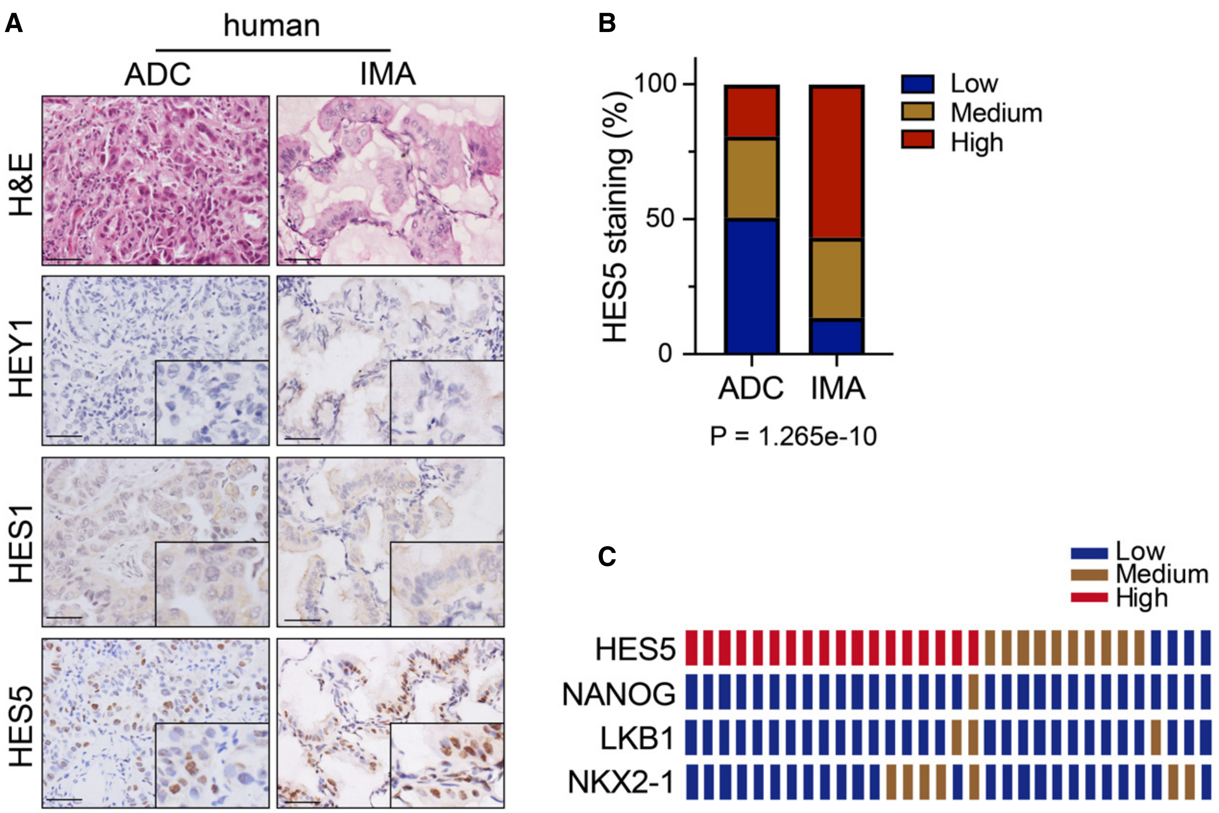

Figure EV4. Increased expression of HES5 in human IMA.

A Representative photographs of H&E and HEY1, HES1, HES5 IHC staining in human ADC and IMA. Scale bar, 50μm.

B Statistic analysis of low, medium, high expression ratio of IHC staining for HES5 in human ADC and IMA. Significance was calculated by χ2 test for trend.

C Expression of HES5, NANOG, LKB1, NKX2-1 in matching IMA tissues (1 row/patient).

 © 2021 The Authors

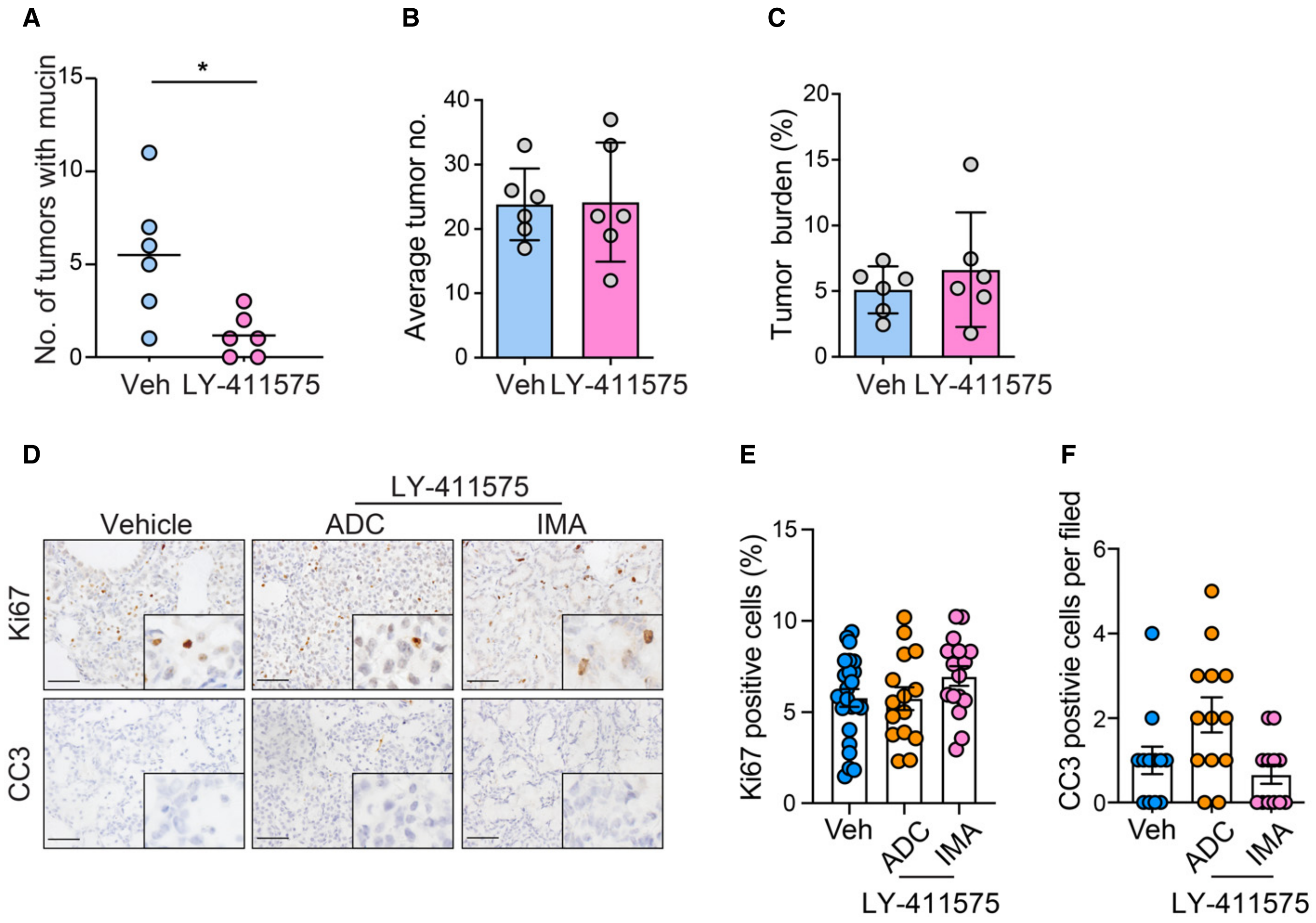

Figure EV5. LY-411575 treatment is insufficient to limit tumor progression in KLN model.

A Quantification of numbers of tumors with mucin in vehicle (n = 6) and LY-411575 (n = 6) groups. Significance was calculated by two-tailed unpaired Student's t-test with Welch's correction. *P = 0.0262

B, C Statistical analysis of total tumor number (B) and tumor burden (C) in vehicle and LY-411575 groups.

D Representative photographs of IHC staining of Ki67 and CC3 reactivity in ADC and IMA lung sections of KLN mice following vehicle or LY-411575 treatment. Scale bar, 50 μm.

E, F Statistical analysis of Ki67 (E) and CC3 (F) reactivity in ADC and IMA lung sections of vehicle and LY-411575 groups. The number of tumors analyzed over vehicle group and LY-411575 group for ADC and IMA is 23, 15, 16 for Ki67, 12,13, 12 for CC3. Results were shown as mean ± SEM.
